# Tunable stiffness of graphene oxide/polyacrylamide composite scaffolds regulates cytoskeleton assembly†
†Electronic supplementary information (ESI) available: Experimental procedures, characterization of GO sheets and RT-qPCR data. See DOI: 10.1039/c8sc02100g


**DOI:** 10.1039/c8sc02100g

**Published:** 2018-07-02

**Authors:** Yupeng Sun, Kaixiang Zhang, Ruijie Deng, Xiaojun Ren, Can Wu, Jinghong Li

**Affiliations:** a Department of Chemistry , Key Laboratory of Bioorganic Phosphorus Chemistry & Chemical Biology , Tsinghua University , Beijing 100084 , China . Email: jhli@mail.tsinghua.edu.cn

## Abstract

The stiffness of the extracellular matrix (ECM) not only provides mechanical resistance to support the cellular shape, but also plays significant roles in many cell functions. However, it's difficult to utilize traditional substrate materials to investigate cell behaviors under physical microenvironments due to their unphysiological stiffness or intrinsic secondary effects. Herein, a stiffness-tunable graphene oxide/polyacrylamide composite scaffold was fabricated to investigate the effect of substrate stiffness on cytoskeleton assembly and specific gene expression during cell growth. In the composite structure, the polyacrylamide (PAAm) hydrogel plays an exceptional role in controlling the substrate stiffness; in contrast, graphene oxide (GO) sheets not only provide permissive surfaces for cell adhesion and growth, but also effectively eliminate the secondary effects of the PAAm hydrogel. It's found that substrate stiffness could affect cell morphology and cytoskeleton assembly *via* specific genetic pathways. Therefore, the composite structure can be considered an attractive candidate as a scaffold and provides potential to elucidate the disease association of ECMs.

## Abstract 2

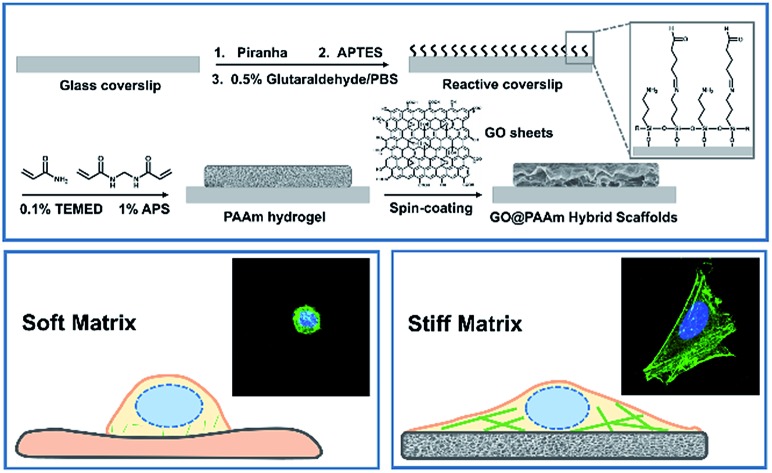
Graphene oxide/polyacrylamide composite scaffolds with tunable stiffness are designed and fabricated to investigate the effect of extracellular matrix (ECM) stiffness on cytoskeleton assembly and specific gene expression during cell growth.

## Introduction

Cell fate is known to be regulated by signals from the microenvironment,^1^ not only through soluble signals but also through biophysical cues.^2^–^5^ Biophysical cues, including topographical features, geometries and substrate stiffness, control upstream cytoskeleton assembly and downstream gene expression *via* a mechanotransduction pathway,^6^ which can ultimately modulate cellular behaviors, such as cell spreading, migration, proliferation and differentiation.^7^–^9^

Recent literature suggests that substrate stiffness can affect cell adhesion and consequently cell growth by changing the cell shape and actin cytoskeleton, and meanwhile demonstrates key roles in cell signaling and differentiation.^10^,^11^ A traditional cell-culture dish, such as polystyrene and glass, is used for unphysiologically stiff materials, and cells cultured on these substrates tend to display aberrant behaviors: anomalous polarization, flattened shapes and loss of differentiated phenotypes.^12^ Thus, it's crucial for a desirable biomaterial to own distinct properties including biocompatibility and the ability to support appropriate cellular growth and function, especially tunable stiffness similar to native tissues.

Hydrogels have provided a useful platform to reveal fundamental phenomena, regulate cell behavior and direct stem cell differentiation in ways not possible with conventional culture substrates.^13^ However, the fabrication of hydrogel-based substrates is an extremely complicated, expensive and skilled process. Besides, it may produce secondary effects of hydrogels, that is to say, changes in hydrogel stiffness could lead to alteration of the density of cell-adhesive ligands or the porosity of the underlying scaffold. A lower collagen anchoring density and larger anchoring distance was demonstrated to result in increased differentiation.^14^ But Engler's group claimed that the stiffness of planar matrices regulated stem cell differentiation independent of protein tethering and porosity by modulating substrate porosity without altering stiffness in polyacrylamide (PAAm) hydrogels.^15^ Thus, there is still a debate whether stiffness or the secondary effects control cell function.

To address this issue, a graphene oxide (GO) sheet, which is a single layer of sp^2^ hybridized carbon atoms tightly packed into a two-dimensional (2D) honeycomb lattice with oxygen-containing hydrophilic groups, was taken into consideration.^16^ We hypothesize that GO can act as a biocompatible coating material to generate homogeneous topographical features in the surface of GO-coated PAAm hydrogels with different stiffnesses. This GO/PAAm composite scaffold will have the following advantages: (i) no secondary effects as GO covered the surface of PAAm hydrogels, (ii) the tunable stiffness ability, (iii) biocompatibility (GO has been widely regarded as a very low cytotoxicity material).^17^,^18^

Herein, we report a simple, practicable and cost-effective strategy to regulate cell behaviors and functions *via* a tunable stiffness enabled GO/PAAm composite scaffold. Benefited from the biocompatibility and the capability for blocking the secondary effects of PAAm hydrogels, the GO/PAAm composite structure can mimic the stiffness of native tissues to investigate the influence of substrate stiffness on cell behaviors and gene expression during cell growth. It's found that the tunable stiffness of the GO/PAAm composite scaffold could affect cytoskeleton assembly, morphology and the expression of cellular signal regulation and cytoskeleton-related genes. Thus, this GO/PAAm composite scaffold can be regarded as an alternative biomaterial to investigate the deep molecular mechanism for the influence of mechanical cues on cell growth in their physical microenvironments.

## Results and discussion

### Overview of GO/PAAm composite scaffolds

**Scheme 1:**
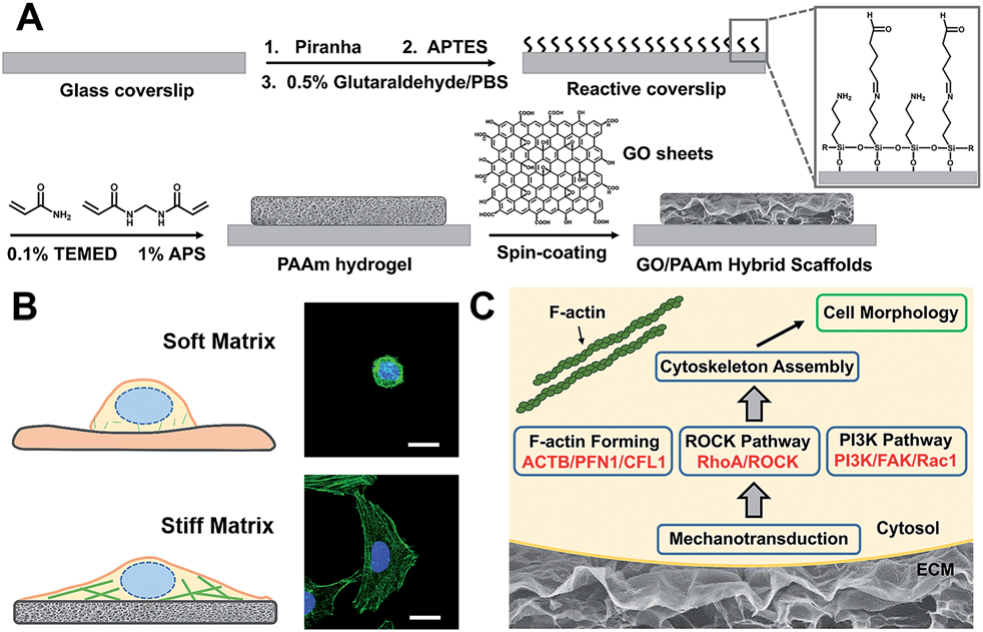
Schematic illustration of cell behaviors on the GO/PAAm composite hydrogel matrix with tunable stiffness. (A) Schematic illustration of the fabrication process of the proposed GO/PAAm composite scaffolds. (B) Schematic and fluorescence images of the cell morphology of the soft and stiff substrates. (C) Hypothetic gene pathways associated with cellular cytoskeleton assembly and cell morphology.

Scheme 1 illustrates the processes employed for the fabrication of the stiffness-tunable GO/PAAm composite scaffolds and the effect of substrate stiffness on cell growth. As illustrated in Scheme 1A, to make the stiffness-tunable GO/PAAm composite scaffolds a substrate for cell growth, piranha-cleaned glass was modified with amine groups by using 3-aminopropyltriethoxysilane (APTES), and then treated with 0.5% glutaraldehyde/PBS to generate a reactive surface. By varying the amounts and ratios of monomers acrylamide and cross-linker bis-acrylamide, the stiffness-tunable PAAm hydrogels were synthesized. Considering the 2D flexible structure and the biocompatibility of GO sheets, we demonstrate the use of GO sheets as an effective coating material in combination with the stiffness-tunable PAAm hydrogels for investigating the influence of substrate stiffness on cell growth.

A substrate stiffness-dependent cellular behavior is presented in Scheme 1B. Briefly, the cells cultured on the soft matrix present a round shape, while the cells on the stiff matrix show a spindle shape and obvious stress fibers. Scheme 1C illustrates the hypothetical gene pathways associated with cytoskeleton assembly. The cells transfer the extracellular stiffness information into different intracellular gene pathways related to cytoskeletal rearrangement *via* mechanotransduction, and then these signaling pathways induce a specific gene expression change, form actin filaments and promote cytoskeleton assembly and cell growth.

### Fabrication of GO/PAAm composite scaffolds with tunable stiffness

**Fig. 1:**
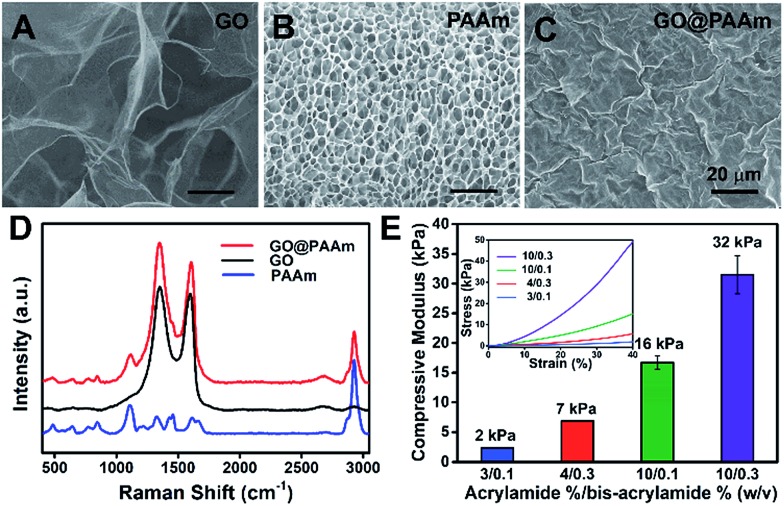
Characterization of the GO/PAAm composite hydrogel matrix. (A–C) Typical SEM images of GO, PAAm hydrogels (10/0.3) and GO/PAAm composite scaffolds. Scale bars, 20 μm. (D) Raman spectra profiles of PAAm hydrogels (blue), GO (black) and the as-prepared GO/PAAm composite scaffolds (red). (E) Mechanical properties of GO/PAAm composite scaffolds with different monomer-to-crosslinker ratios. Error bars represent the SD of measurements performed on three samples. The inset is the representative stress–strain curves.

To fabricate the stiffness-tunable GO/PAAm composite scaffolds, we integrated the PAAm hydrogels with GO sheets *via* a spin-coating method. As shown in Fig. 1A, flexible and large-size GO sheets were synthesized by a modified Hummers' method.^19^,^20^ The GO sheets displayed a large lateral dimension of over 30 μm with a typical wrinkled topography (Fig. S1A–C†) and had a typical thickness (∼1.58 nm) of sub-bilayer GO sheets (Fig. S1D and E†). In contrast, the surface of the freeze-dried PAAm hydrogel presented a multiporous network structure with uniformly distributed pore sizes of almost 5 μm (Fig. 1B). It is precisely the gel porosity and its variance in the hydrogels of differing stiffness that result in the secondary effects. Fortunately, this issue was simply and effectively addressed *via* a GO-coated PAAm hydrogel composite structure, in which the flexible and large-size GO sheets covered the porous structure on the PAAm hydrogel surface, formed a homogeneous GO film with a mildly wrinkled topography and eliminated the secondary effects that resulted from the gel porosity (Fig. 1C). To obtain a suitable GO concentration for an effective coating, the GO dispersions in a range of 0.005 to 0.5 mg ml^–1^ were dispersed in water and ultrasonically treated for an hour before coating. From the SEM images of the GO/PAAm composite structures (Fig. S2†), we could see that as the concentration of GO dispersions increases, the multiporous surface of the PAAm hydrogel was gradually covered with GO sheets. Finally, we chose 0.1 mg ml^–1^ as an ideal concentration; in this case, GO sheets not only were effectively coated onto the PAAm hydrogels, but also were tightly bound to the surface of the PAAm hydrogel and hard to exfoliate. The stability of GO/PAAm composite structures benefits from the electrostatic interaction between PAAm hydrogels (positive) and GO sheets (negative).^21^ As shown in Fig. 1D, the Raman spectra of the GO/PAAm composite scaffolds (red) showed the existence of the two characteristic peaks of the D band and G band of GO sheets (black), and at the same time, presented the characteristic peaks of the PAAm hydrogel (blue) at 1112 cm^–1^ and 2930 cm^–1^, indicating the formation of GO/PAAm composite scaffolds.^22^ Furthermore, the as-prepared GO/PAAm composite structure was also confirmed by using FT-IR spectra (Fig. S3†).

To simulate the stiffness of different human organs, 4 formulations of monomer-to-crosslinker (acrylamide/bis-acrylamide) were polymerized to yield PAAm hydrogels of ∼2, ∼7, ∼16 and ∼32 kPa, which correspond to the stiffness of the brain tissue, adipose tissue, muscles and osteoid, respectively.^23^–^25^ The compressive modulus (namely the stiffness) is a key physical property for the GO/PAAm composite scaffolds, which contribute to cell adhesion and growth.^26^,^27^ The hydrogel stiffness was controlled by the ratio of acrylamide and bis-acrylamide and the details are supplied in Table S1.† The different hydrogels revealed significant differences in mass swelling ratios, stiffness and surface texture (or porosity). When coated with an optimum concentration of GO sheets, the composite scaffolds showed a typical topography similar to that of GO thin films (Fig. S4†) and the stress–strain curves of PAAm hydrogels and GO/PAAm composite scaffolds are also analogical (Fig. S5†). As shown in Fig. 1E, the compressive modulus of the composite scaffold for a monomer-to-crosslinker ratio of 10/0.3 was significantly higher than that of 3/0.1, and as the ratio of acrylamide/bis-acrylamide increases, the compressive modulus increased from 2 kPa to 32 kPa. Together, the GO/PAAm composite scaffolds not only could simulate the stiffness of native tissues, but also owned a uniform surface appearance to eliminate the intrinsic secondary effects in hydrogels. Therefore, the GO/PAAm composite scaffolds will provide a powerful platform for investigating cell behaviors related to the substrate stiffness.

### The influence of ECM stiffness on cytoskeleton assembly and cell morphology

**Fig. 2:**
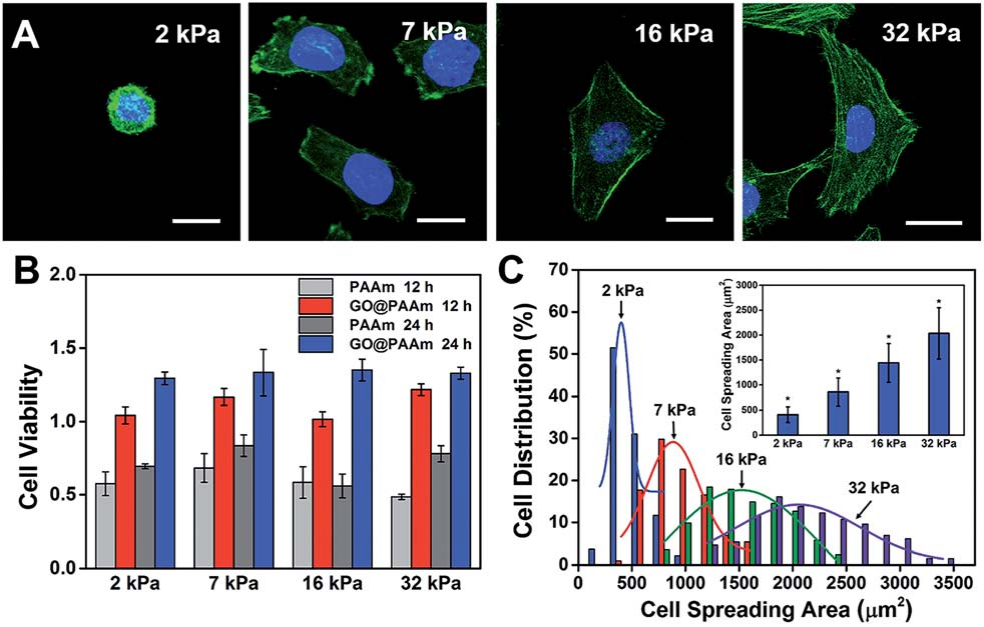
Cell behaviors on the GO/PAAm composite hydrogel matrix with different stiffnesses. (A) Fluorescence images of HeLa cells on the substrates with varied stiffness: 2 kPa, 7 kPa, 16 kPa, and 32 kPa. Scale bar, 20 μm. (B) Cell viability is measured by a CCK-8 test after incubation on different substrates as indicated for 12 and 24 h. The results have been normalized to HeLa viability on a tissue culture plate. (C) The distribution of the cell spreading area after seeding on different substrates for 24 h; the inset shows the average spreading area on different substrates (mean ± s.d.; *P < 0.05).

The biocompatibility of scaffold materials is essential for cell survival and function in engineered tissues.^28^ To evaluate cellular proliferation cultured on the GO/PAAm composite scaffolds, a cell counting kit (CCK) assay was employed to measure the cell viability during the culture period (Fig. 2B). For the first 12 h, cells cultured on the GO/PAAm composite scaffolds (red columns) appeared to support cellular growth with a higher cell viability compared to bare PAAm hydrogels (light grey columns), as in the case of the cell cultured for 24 h. By contrast, for 12 and 24 h, the cell viability increased, independent of the substrate stiffness in all groups including the GO/PAAm composite scaffolds and the bare PAAm hydrogels. Besides, the composite scaffolds showed no significant difference in cell viability from 2 kPa to 32 kPa for cells incubated for 12 or 24 h. The abnormal and wrinkled state for cells on bare PAAm hydrogels implied high cytotoxicity of the PAAm hydrogel (Fig. S6†). Compared to the traditional PAAm hydrogel coated with collagen, cells cultured on the GO/PAAm composite scaffold presented a similar cell morphology and spreading area, indicating the excellent biocompatibility of the composite structure (Fig. S7†). Thus, the GO/PAAm composite scaffolds displayed better biocompatibility than bare PAAm hydrogels and the stiffness of the substrate had no significant influence on the cell viability.

To investigate the influence of ECM stiffness on cytoskeleton assembly and cell morphology, HeLa cells were cultured on GO/PAAm composite scaffolds with varied stiffness. When incubated on the scaffolds for 24 h, the cells were fixed and stained with Alexa 488-conjugated phalloidin and DAPI, to reveal the actin filament network (green) and the nuclei (blue), respectively. The representative fluorescence images of HeLa cells are shown in Fig. 2A; obvious stress fibers (filamentous actin bundles) were seen in cells grown on the stiffer matrix (32 kPa), but not in cells grown on the softer matrix (2 kPa, 7 kPa or 16 kPa). Stress fibers, which play an important role in cellular cytoskeleton assembly, can provide force for cells to sense and transmit the signal of the ECM stiffness.^29^ As shown in Fig. S8,† it is confirmed that cells grown on stiffer GO/PAAm composite scaffolds (optical image) presented stress fibers (fluorescence images), and for the softer scaffolds, the cells showed a smaller shape and no obvious stress fibers were seen. Next, to further quantify the relationship between cell morphology and matrix stiffness, the cell spreading area was taken into statistical analysis. As presented in Fig. 2C, the cell spreading area demonstrated normal distribution characteristics, and as the matrix stiffness increases, the shift of distributions suggested increase of cell spreading area, and the distribution was getting wider. Typically, the cells on the soft substrate (2 kPa) presented a round shape and smaller spreading area (400 μm^2^), while the cells cultured on the stiff substrate (32 kPa) showed a spindle shape and larger spreading area (2000 μm^2^) (Fig. 2C). Therefore, the morphological analysis suggests that the stiffness of GO/PAAm composite scaffolds would significantly affect the cytoskeleton assembly, shape and spreading.

### The role of the PAAm hydrogel in the GO/PAAm composite structure

**Fig. 3:**
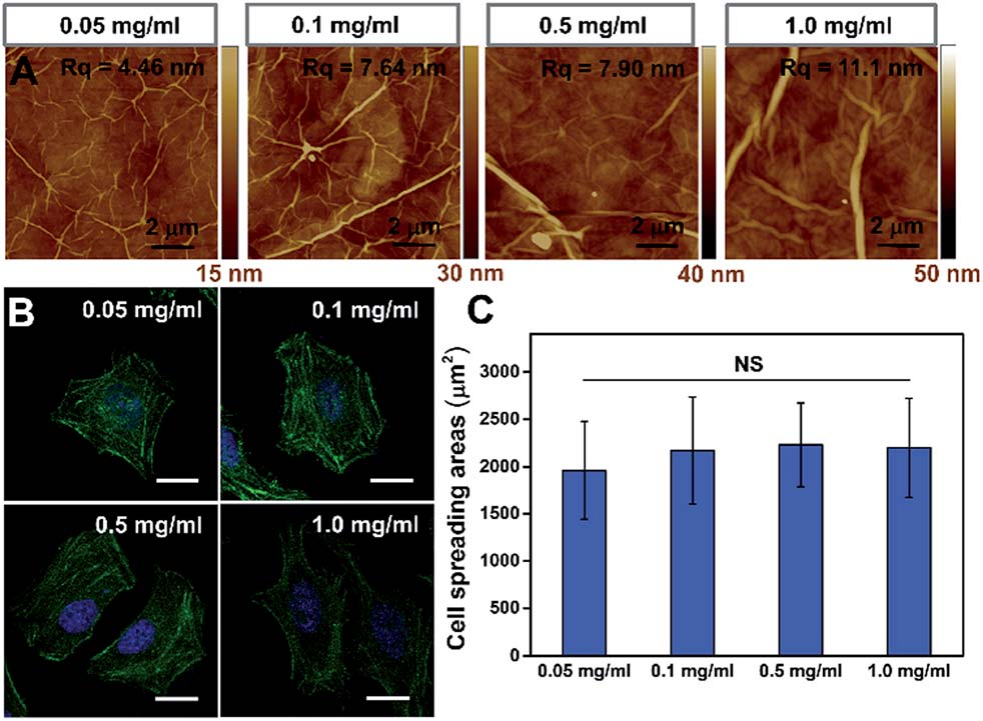
The influence of the GO film matrix on cell behavior. (A) Tapping mode AFM height of GO films fabricated with different concentrations of GO dispersions: 0.05 mg ml^–1^, 0.1 mg ml^–1^, 0.5 mg ml^–1^ and 1.0 mg ml^–1^. (B) Fluorescence images of HeLa cells on the GO films fabricated with varied GO concentrations (as indicated). Scale bar, 20 μm. (C) Statistical analysis of the cell spreading area on different GO films. NS indicates that the difference is not statistically significant by Student's *t*-test.

To further confirm that the PAAm hydrogel plays an important role in the GO/PAAm composite structure for guiding cell behaviors, we investigated the effect of cell growth in the case of only GO films. Fig. 3A shows the typical topographical features of GO films with concentrations in the 0.05–1.0 mg ml^–1^ range. The surface root-mean-square roughness (*R*_q_) of the GO films were 4.46 nm, 7.64 nm, 7.90 nm and 11.1 nm, respectively. For the GO film fabricated using 0.05 mg ml^–1^ GO solution, we observed a very thin, continuous and uniform GO film with an appearance of micrometer-long wrinkles. With the GO concentration increasing, we observed an increased disorder and roughness, and long, broad wrinkles were observed across the film surface. As shown in Fig. 3B, cells cultured on the different GO films showed no significant difference in cellular morphology. Besides, we found that it displays no remarkable difference of stress fibers and cell spreading area for cells under different GO films (Fig. 3C), though the roughness of these GO film surfaces showed differences. That is to say, there was no significant effect on cell morphology, despite some mild changes in the topographical features of the substrate surface.^30^,^31^ However, for the substrate stiffness, the case is completely different. The stiffness of substrates not only regulated the cellular morphology, but also affected cytoskeleton assembly (the formation of stress fibers). Therefore, the tunable stiffness of the PAAm hydrogel plays a central role in GO/PAAm composite scaffolds, providing the ability to regulate cell behaviors. Also GO sheets are very important, and enhance the biocompatibility and address the issue of secondary effects due to the different surface structures of the PAAm hydrogel.

### The role of gene expression programs in cytoskeleton assembly

**Fig. 4:**
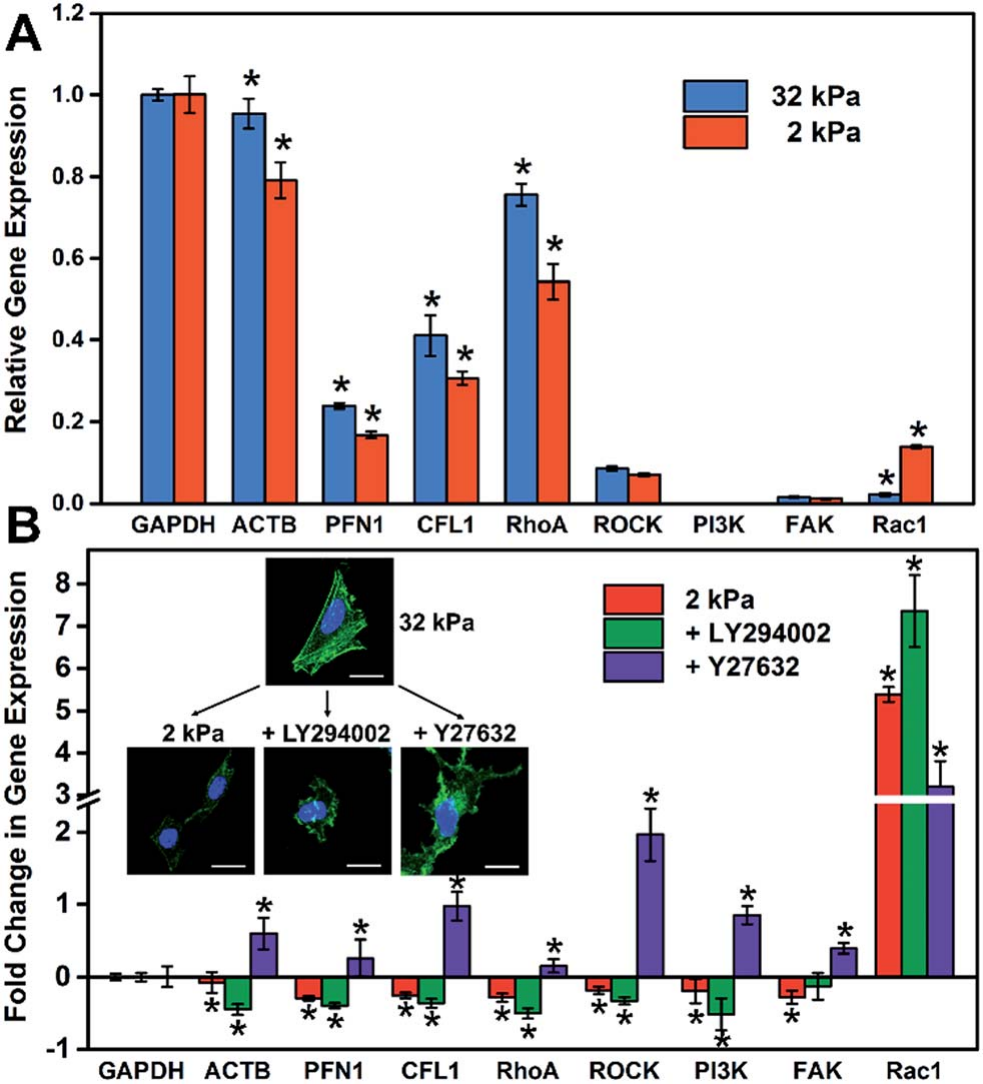
The relationship between the stiffness of the GO/PAAm composite matrix and cellular gene expression. (A) Expression profiles of cell adhesion- and cell spreading-associated genes on the soft (2 kPa) and the stiff (32 kPa) matrix. (B) Fold change in gene expression under different stimulations: soft matrix (2 kPa); LY294002 (inhibiting PI3K); Y27632 (inhibiting ROCK). The inset is the fluorescence images of HeLa cells that display the cell morphology under different stimulations. The gene expression is normalized to the stiff matrix relative to GAPDH. Student's *t*-test was used for evaluating the significance (*P < 0.05).

It is well known that gene expression programs and cell signaling pathways play important roles in regulating cell functions.^32^ For instance, Steven and Andre suggested that the effect of the matrix stiffness on cell directed migration depended on the balance of the ECM-triggered signaling pathways PI(3)K and ROCK.^33^ To investigate the relationship between the substrate stiffness and cell specific gene expressions, RT-qPCR was employed to analyze the expression of cytoskeleton-related genes (ACTB, PFN1 and CFL1) and cell signaling-related genes, such as the ROCK pathway (RhoA and ROCK) and PI3K pathway (PI3K, FAK and Rac1). These genes regulated or participated in cytoskeleton assembly, which is an important process for the formation of stress fibers and cell spreading.^34^–^37^ As shown in Fig. 4A, the expression level of cytoskeleton-related genes (ACTB, PFN1 and CFL1) on the soft matrix presented a significant reduction compared to the stiff matrix, indicating a lower efficiency or depletion in number to produce stress fibers, because these genes participated in cytoskeleton assembly. Therefore, cells grown on the stiff matrix presented obvious stress fibers, but not for cells on the soft one (consistent with the experiment above). On the other hand, the expression level of RhoA and cell signaling-related genes decreased and Rac1 increased distinctly on the soft matrix compared to the stiff matrix, indicating that matrix stiffness could regulate gene expression programs, and besides RhoA and Rac1 may play different roles in cytoskeleton assembly and cell morphology.

To further investigate how the matrix stiffness affects cytoskeleton assembly and cell morphology *via* regulating specific gene expression programs, HeLa cells were cultured on stiff and soft matrixes, and the cells on the stiff matrix were treated with a panel of pathway and protein inhibitors, including Rho-associated kinase (Y-27632) and PI3K (LY-294002) (RT-qPCR data are shown in Fig. S9†). As shown in Fig. 4B, the fold change in gene expression for the soft group is consistent with the group added PI3K inhibitor, and almost opposite with the group added ROCK inhibitor except for Rac1. This phenomenon may suggest that the PI3K pathway for cells on the soft matrix was restrained through matrix stiffness-related gene regulatory processes. To more rigorously verify the findings, cell morphology under different stimulations was taken into consideration (Fig. 4B), and the cell spreading area was significantly reduced in the presence of inhibitors of PI3K (LY-294002) and the cell presented a corrugated state as the stress fibers disappeared, which is consistent with the case for the cell on the soft matrix. In contrast, when inhibiting Rho-associated kinase with Y-27632, there was no distinct change in cell spreading area, but the cell also couldn't form stress fibers resulting in an unconsolidated cell morphology. Together, all of this confirms the role of the PI3K and the ROCK pathway in mediating the formation of stress fibers, and the cell morphology is also relative to the PI3K pathway. Mechanical cues from the microenvironment regulate cell behaviors during growth *via* gene-regulatory processes, in which specific gene expression programs are activated and induce cytoskeleton assembly, promoting the formation of stress fibers and cell spreading. The GO/PAAm composite scaffold with tunable stiffness has served as a powerful platform for investigating the underlying mechanisms of how substrate stiffness regulates cytoskeleton assembly under physical microenvironments.

## Conclusions

In summary, we have demonstrated the capability of a unique GO/PAAm composite scaffold to provide instructive physical cues that regulate specific gene expression programs and cell behaviors, such as cytoskeleton assembly and cell morphology. For the composite scaffold, the PAAm hydrogel plays an exceptional role in controlling the substrate stiffness; in contrast, GO sheets not only provide permissive surfaces for cell adhesion and growth, but also effectively reduce the secondary effects utilizing their uniform surface topographical features. It's found that cytoskeleton assembly and cell morphology can efficiently be regulated by the substrate stiffness. And cytoskeleton-related genes (ACTB, PFN1 and CFL1) participated in cytoskeleton assembly, and the PI3K and ROCK pathways play important roles in mediating the formation of stress fibers. We envision that these findings can help in understanding the molecular mechanism for the influence of ECM mechanical cues on cell growth and metastasis at a deeper level. Furthermore, such a GO/PAAm composite scaffold can serve as a powerful platform for developing future therapies for ECM defect-related diseases and injuries.

## Experimental section

### Preparation of the GO/PAAm hydrogel composite scaffold

Glass coverslips were cleaned with Piranha solution (H_2_SO_4_ : H_2_O_2_ = 3 : 1), and then modified using 3-(trimethoxysilyl)propyl methacrylate (APTES) and glutaraldehyde to facilitate covalent attachment of hydrogel substrates to the amino-silanated coverslips. The preparation of PAAm hydrogels was adapted from a previously described protocol with some minor modifications. Briefly, after the diluted suspension was bubbled with nitrogen gas for at least 15 min to remove oxygen, designated amounts of acrylamide monomers, cross-linker bis-acrylamide, tetramethylethylenediamine (TEMED) and ammonium persulphate were prepared in the PAAm hydrogel with varied stiffness. The ratio of acrylamide and bis-acrylamide and the final concentrations were varied to control the mechanical properties and porosity of the hydrogel (details in ESI Table S1†). The gel solution was sandwiched between the functionalized coverslip and a chloro-silanated glass slide to ensure easy detachment of hydrogels. GO was dispersed in deionized water at varying concentrations (0.005, 0.01, 0.05, 0.1, 0.5 and 1.0 mg ml^–1^). The substrates (cover glass or the PAAm hydrogel with different stiffnesses) were dipped with the GO dispersions directly on top of the substrate for 30 min, followed by spin-coating with 800 rpm for 30 seconds.

### Cell culture

HeLa cells were cultured in standard Dulbecco's modified Eagle's medium (DMEM) with 10% fetal bovine serum, 1% penicillin/streptomycin at 37 °C, 5% CO_2_ and 95% air humidity. The GO/PAAm hydrogel substrates with varied stiffness were immersed in PBS and placed in the cell culture hood for 30 min under UV light for sterilization before cell seeding. For cell seeding, HeLa cells were seeded at a proper density on the substrates modified with GO, PAAm hydrogels or the GO/PAAm composite scaffold, so that they had enough space to spread and didn't contact other cells.

### Biocompatibility test

The biocompatibility of the various substrates with varied stiffness (the PAAm hydrogels and GO/PAAm composite scaffold substrate) was tested by examining the cell viability of HeLa cells using a cell counting kit-8 (CCK-8).^38^ HeLa cells were incubated on each substrate for 12 and 24 h in 96-well plates. Following incubation, 100 μL of the cell culture medium (containing 10 μL CCK-8 solution) was added to each well and incubated with cells for 120 min at 37 °C. The data are represented as the absorbance at 450 nm, considering the cells cultured in a well with CCK-8 as the control (*A*_c_), the cells cultured on the substrates with CCK-8 as the experiment group (*A*_s_), and the well with only CCK-8 added as the blank (*A*_b_). Finally, the cell viability is calculated as (*A*_s_ – *A*_b_)/(*A*_c_ – *A*_b_).

### Cell staining and image analysis

After incubation for 24 h and brief washing with sterilized PBS, HeLa cells were fixed in 4% (w/v) paraformaldehyde in phosphate buffered saline (PBS) for 15 min at room temperature (25 °C), permeabilized for 5 min with 0.5% v/v Triton-X100 in PBS at room temperature, and then blocked with 1% BSA for 1 h for actin filament staining. Actin staining was performed using FITC conjugated to phalloidin. After post-stain washing with PBS, the cells were mounted in 4′,6-diamidino-2-phenylindole (DAPI) for nuclear staining. For measurements of the cell-spreading area, the 2D images of phalloidin/DAPI-stained cells were taken using a Leica TCS SP5 inverted confocal microscope (Leica, Germany) with a 63× oil-immersion objective. Only those cells that did not exhibit any cell–cell contacts were considered in the analysis. The boundaries of all single cells were then outlined manually on the basis of the actin stain, and the cell spreading area was determined using Image J software.

### Real-time quantitative PCR (RT-qPCR) analysis

HeLa cells were harvested after counting of cells and the total RNA was extracted by using the TransZol reagent following the manufacturer's instructions. Total RNA samples were investigated on a NanoDrop spectrometer (ND_200, NanoDrop Technologies, USA). RT-qPCR analysis of mRNA was performed with SYBR Select Master Mix according to the manufacturer's instructions on a Bio-Rad C1000TM (Bio-Rad, USA). The primers used for RT-qPCR are listed in Table S2.† Briefly, for this PCR, the 20 μL reaction solution contained 2 μL of cDNA sample, 10 μL 2× SYBR Select Master Mix, 2 μL forward primer (5 μM), 2 μL reverse primer (5 μM), and 4 μL RNase-free water. The total PCR volume was 20 μL and the PCR was carried out for 2 min at 95 °C, followed by cycling 45 × (95 °C for 15 s and 60 °C for 1 min), and finished with 60 °C for 5 min. The experiment was repeated three times. The relative expression level of target mRNAs was evaluated by referring to the expression of GAPDH mRNA using the 2^–ΔΔ*C*_t_^ method.^39^

### Inhibition experiments

For drug inhibition experiments, drug inhibitors were added at 24 h of culture, and sequentially incubated for another 24 h for cell staining and the RT-qPCR experiment. The concentration of the inhibitor used was 20 μM for LY294002 (PI3K inhibitor) and 10 μM for Y27632 (ROCK inhibitor).

## Conflicts of interest

There are no conflicts to declare.

## Supplementary Material

Supplementary information
