# Supplementary material for: Tunable stiffness of graphene oxide/polyacrylamide composite scaffolds regulates cytoskeleton assembly
Source: Chem Sci. 2018 Jul 2;9(31):6516–22. doi: 10.1039/c8sc02100g (PMC6115675; doi:10.1039/c8sc02100g)
Supplement: Supplementary file 1 [file SC-009-C8SC02100G-s001.pdf]

Electronic Supplementary Material (ESI) for Chemical Science.
This journal is © The Royal Society of Chemistry 2018

Supporting Information

Tunable Stiffness of Graphene Oxide/Polyacrylamide Composite Scaffolds Regulates Cytoskeleton Assembly

Yupeng Sun, Kaixiang Zhang, Ruijie Deng, Xiaojun Ren, Can Wu and Jinghong Li*

Department of Chemistry, Key Laboratory of Bioorganic Phosphorus Chemistry & Chemical Biology, Tsinghua University, Beijing 100084, China.
E-mail: jhli@mail.tsinghua.edu.cn.

Table of Contents

1. Experimental procedures. S2
2. Table S1. Mechanical and swelling properties of PAAm hydrogels and GO/PAAm composite scaffolds after polymerization of relative acrylamide and bis-acrylamide concentration. S5
3. Table S2. Primers used for RT-qPCR. S6
4. Figure S1. TEM, SEM and AFM images of GO sheets. S7
5. Figure S2. SEM images of the GO/PAAm composite scaffolds fabricated with different concentrations of GO dispersions. S8
6. Figure S3. FT-IR spectra profiles of PAAm hydrogels, GO and the as-prepared GO/PAAm composite scaffolds. S9
7. Figure S4. Fabrication of stiffness-tunable GO/PAAm composite scaffolds. S10
8. Figure S5. Stress-strain curves of PAAm hydrogels and GO/PAAm composite scaffolds. S11
9. Figure S6. SEM images of HeLa cells on different substrates for 24h. S12
10. Figure S7. Fluorescence images and optical images of HeLa cells on the substrates with varied stiffness. S13
11. Figure S8. The surface morphology and cell behaviors for traditional collagen coated PAAm hydrogel and GO/PAAm composite scaffold. S14
12. Figure S9. Expression analysis of target mRNAs in the HeLa cells under different stimulations by RT-qPCR. S15

Experimental procedures

Materials

Natural graphite flakes (~200 mesh) were provided by Qingdao Tianhe Graphite Co. Ltd (Qingdao, China). Sulfuric acid, hydrochloric acid, ethyl alcohol, Potassium permanganate, Hydrogen peroxide (30% in water) and sodium nitrate were purchased from Sinopharm Chemical Reagent Co., Ltd. Triton-X100, 4% paraformaldehyde in PBS buffer, DPBS, fetal bovine serum, Dulbecco's modified eagle medium (DMEM), Trypsin and Penicillin-Streptomycin were purchased from Beijing Solarbio Science & Technology Co., Ltd. (Beijing, China). TransZol, RevertAid First Strand cDNA Synthesis Kit and SYBR select master mix were bought from Thermo Fisher Scientific (Waltham, USA). Glass coverslips (12 mm) were purchased from VWR (West Chester, USA). FITC-phalloidin, DAPI, Acrylamide and bis-acrylamide were purchased from Sigma-Aldrich (St. Louis, USA). Ammonium persulphate (APS), N,N,N',N'-Tetramethylethylenediamine (TEMED), glutaraldehyde (~25% in H_2O), 3-aminopropyltriethoxysilane (3-APTES) were bought from Beijing DingGuo Biotechnology Co., Ltd. (Beijing, China). Ultra-pure water (18 MΩ CM) was produced by a Millipore System (Millipore Q, USA).

Preparation of Graphene Oxide

Thin-layered GO was prepared by a modified Hummers method. Briefly, graphite (3 g, 200 mesh) was added into 98% H_2SO_4 (70 ml) and $NaNO_3$ (1.5 g) mixed solution, then $KMnO_4$ (9 g) was slowly added into the mixture in a 15 min time period with stirring under the ice bath condition. After 30 min, the temperature of the mixture was gradually raised to 45° C and was stirred for 30 min. Subsequently, de-ionized water (141 ml) was slowly dropped into the mixture with vigorous stirring, and kept at 95℃ for 15 min. Finally, the reaction was quenched by the addition of de-ionized water (360 ml) followed by the addition of 30% H_2O_2 (21 ml). The graphite oxide was filtered with 3% HCl solution (450 ml; three times) and de-

ionized water (50 ml) to get the graphite oxide filter cake. Then, the filter cake was re-dispersed into de-ionized water (2000 ml) with vigorous stirring overnight, followed by dialyzing for 1 week (dialysis bag, 8000-14000 Da; replacing the ionized water, twice). A homogeneous suspension was collected after removing the trace black residues by centrifugation at 3000 rpm for 3 min. After centrifugation of the solution at 10 000 rpm for 30 min, the suspension with single or few-layer graphene oxide was finally obtained. GO powder was obtained after freezing and drying of the suspension.

Characterization of the Substrates

Swelling ratio. Swelling experiments were performed by immersing the hydrogels in distilled water at room temperature overnight. The wet weight of PAAm hydrogels was weighed after removing the excess water from the surface by filter papers. The dry weight was weighed by the freeze dried samples of the PAAm hydrogel. The mass-swelling ratio (Qm) is typically defined as the ratio of wet weight（Mw）to dry weight (Md), at least 3 times per sample were counted for these measurements. *Scanning Electron Microscopy (SEM).* GO dispersions were spin-coated onto the surface of APTES-treated silicon wafer, and dried by blowing nitrogen gas. GO powder was prepared by freeze-drying of GO solutions. The as-prepared GO/PAAm composite scaffold and pure PAAm hydrogel samples were swollen in PBS overnight, then flash-frozen by plunging into liquid nitrogen. The substrates were freeze dried overnight. Subsequently, the lyophilized samples were cut to expose their cross-sections and coated with Pt/Pd using a sputter coater. Cultured HeLa cells on the substrates were washed with PBS (pH 7.4) and fixed in 3% glutaraldehyde in PBS for 1 h. Following by freeze-drying overnight, the lyophilized samples were coated with Pt/Pd using a sputter coater. The detailed structure of the sample cross-sections was imaged using an SEM (SU-8010 HITACHI, Japan). *Transmission electron microscope (TEM).* GO dispersions were dipped onto Micro grid copper net (air-dry). The morphology of GO was observed using a HT7700 TEM at an accelerating voltage of 100 kV. *Atomic Force Microscope (AFM).* GO dispersions were spin-

coated onto the surface of APTES-treated silicon wafer, and dried by blowing nitrogen gas. The atomic force microscopy image and profile of exfoliated graphene oxide sheets were taken with a Bruker Dimension Icon AFM using a silicon probe. *Raman spectroscopy.* Raman analysis of PAAm hydrogels, GO and the as-prepared GO/PAAm composite substrate was carried out using a Horiba-Jobin-Yvon Raman system equipped with an Ar-ion excitation laser (λ = 514 nm, power = 0.5 mW). A 50 x microscope objective was used to focus the laser beam and collect the scattered light. *FTIR Spectroscopy.* Attenuated total reflection Fourier Transform IR spectra (ATR-FTIR) of PAAm hydrogels, GO and the as-prepared GO/PAAm composite substrates were carried out on an UATR Two FT-IR spectrometer (Perkin Elmer, USA). Briefly, the freeze-drying samples were put on the window and the spectra were scanned from 400 cm^{-1} to 4000 cm^{-1}. *Mechanical property measurements.* The samples were incubated in DPBS at room temperature for overnight to reach a fully swollen state. Specifically, a centrifugal tube (1.5 ml) was used to create a circular hydrogel disc with a diameter of 10 mm and a thickness of 4~8 mm, approximatively. The cylindrical hydrogels were incubated in DPBS at room temperature for overnight to reach a fully swollen state. Compression tests of the as-synthesized samples were performed on a model 3342 universal testing machine at a rate of 1% strain/min. The raw force, F(t), and displacement, u(t), time series data were converted into the frequency domain, F(ω) and u(ω), using a fast Fourier transform. The complex modulus of the sample was calculated as: $E = F(\omega)\cdot l/\ u(\omega)\cdot A^{-1}$, where l is the sample height and A is the surface area of the sample. The compressive modulus was calculated from the slope in the linear region corresponding to 5-15% strain. Measurement was performed on 3 replicate samples in each group.

Table S1. Mechanical and swelling properties of PAAm hydrogels and GO/PAAm composite scaffolds after polymerization of relative acrylamide and bis-acrylamide concentration.[a]

Acrylamide (%)	Bis-acrylamide (%)	APS/TEMED (%)	Compressive Modulus (kPa) PAAm	Q_m[b]	Compressive Modulus (kPa) GO/PAAm
3	0.1	1/0.1	2.047±0.01	41.29±3.56	2.375±0.02
4	0.3	1/0.1	6.16±20.08	22.31±1.10	6.884±0.06
10	0.1	1/0.1	16.24±1.32	14.68±0.71	16.70±1.14
10	0.3	1/0.1	32.06±4.08	11.08±0.85	31.51±3.21

[a] This table presents the relative concentrations of acrylamide and bis-acrylamide and their elastic modulus and mass-swelling ratio (Q_m) after polymerization in PBS.

[b] The mass-swelling ratio (Q_m) is typically defined as the ratio of wet weight (M_w) to dry weight (M_d), at least 3 times per sample were statisticsed for these measurements.

Table S2. Primers used for RT-qPCR.

Gene	Forward primer (5`-3`)	Reverse primer (5`-3`)
GAPDH	TATGACAACAGCCTCAAGAT	AGTCCTTCCACGATACCA
ACTB	CCAACCGCGAGAAGATGA	CCAGAGGCGTACAGGGATAG
PFN1	CGAGAGCAGCCCCAGTAGCAGC	ACCAGGACACCCACCTCAGCTG
CFL1	CAAGGAGAGCAAGAAGGAGGAT	GTCCTTGGAGCTGGCATAAAT
RhoA	GGGAGCTAGCCAAGATGAAG	GTACCCAAAAGCGCCAATC
ROCK	TGCATTCCAAGATGATCGTTA	AAGATCTCCACCAGGCATGTA
PI3K	CGCCCCCTTAATCTCTTACA	TGGATGTTCTCCTAACCATCTG
FAK	TCCCTATGGTGAAGGAAGT	TTCTGTGCCATCTCAATCT
Rac1	ATGCAGGCCATCAAGTGTGTGGTG	TTACAACAGCAGGCATTTTCTCTTCC

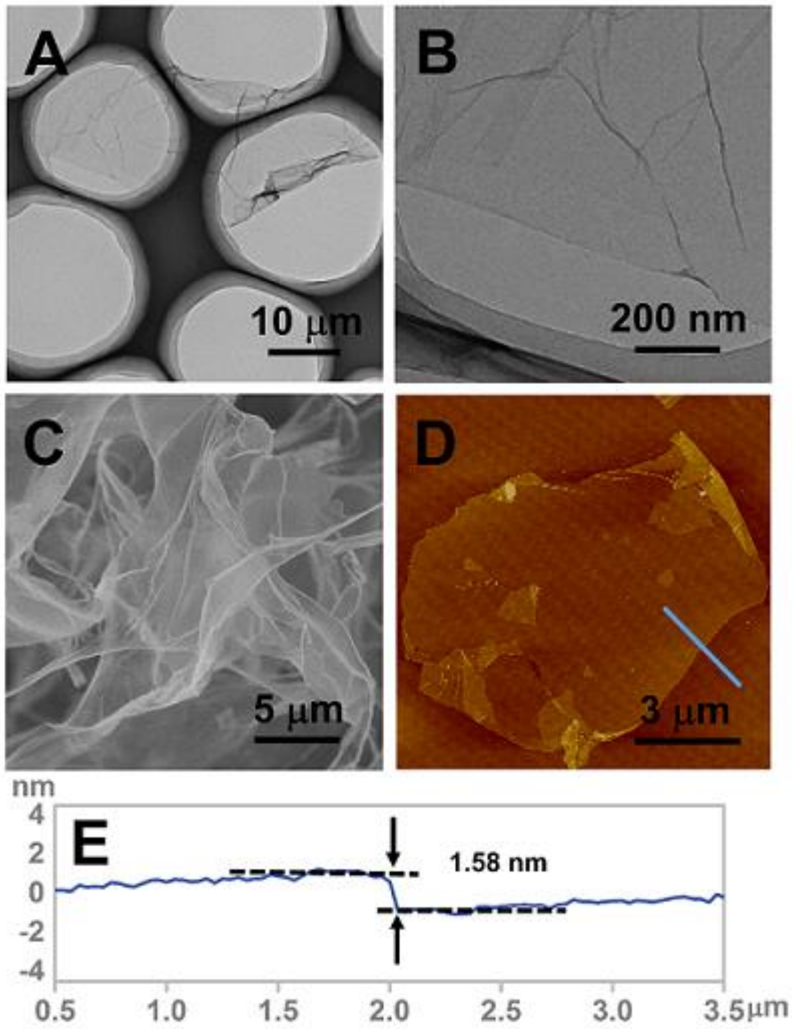

Figure S1. TEM, SEM and AFM images of GO sheets. (A) Low- and (B) high-magnification TEM images showing the layer morphology of GO sheets. (C) A typical SEM image of GO sheets. (D) Tapping mode of AFM image and (E) the thickness of the measured GO sheets is ~1.58 nm.

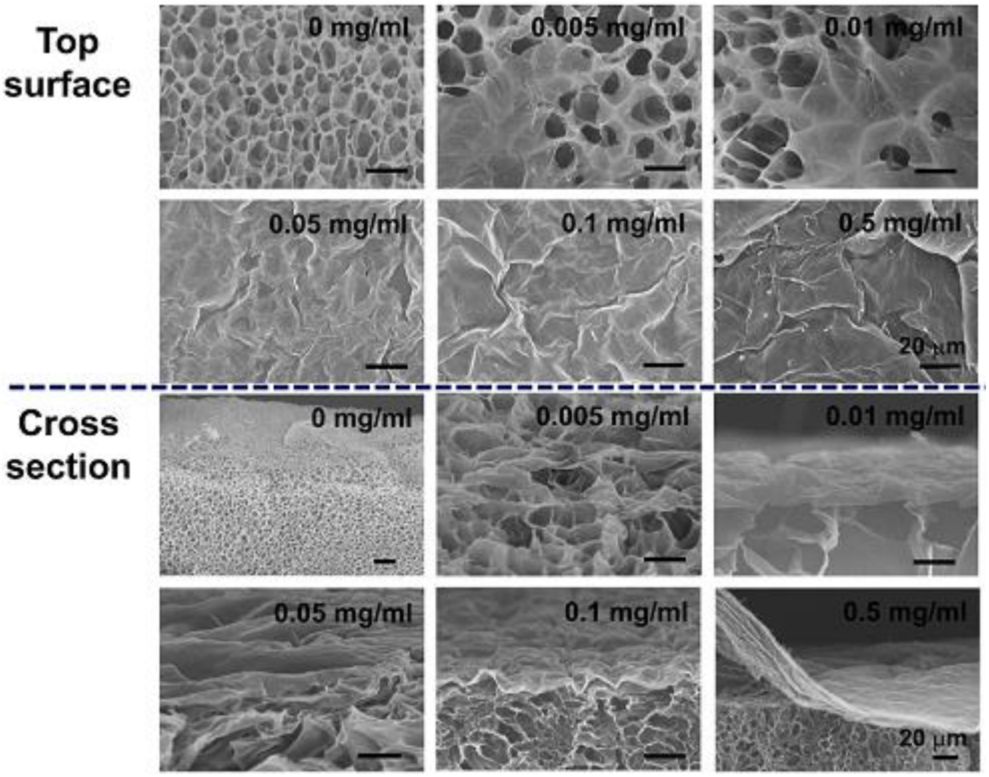

Figure S2. SEM images of the GO/PAAm composite scaffolds fabricated with different concentrations of GO dispersions: 0, 0.005 mg/ml, 0.01 mg/ml, 0.05 mg/ml, 0.1 mg/ml, 0.5 mg/ml (up: top surface; down: cross section). Scale bars, 20 μm.

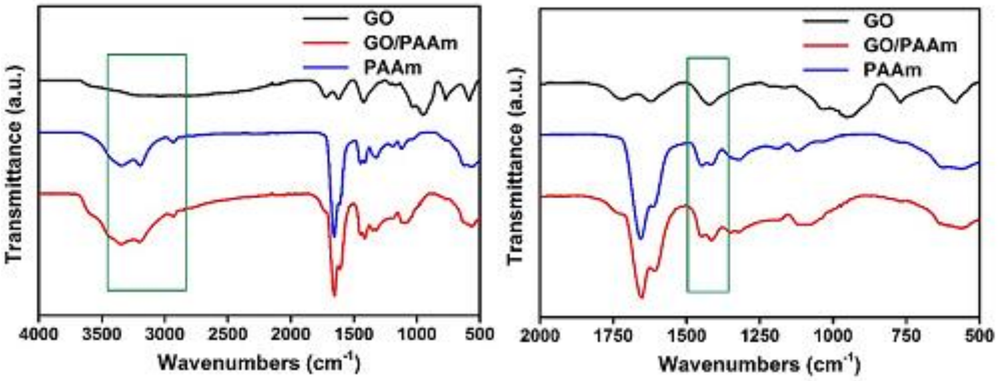

Figure S3. FT-IR spectra profiles of PAAm hydrogels, GO and the as-prepared GO/PAAm composite scaffolds (GO concentration: 0.5 mg/ml).

To further confirm the as-prepared GO/PAAm composite structure, we take FT-IR spectra for illustration in details. As shown in Figure S3, the spectra of GO/PAAm composite scaffolds (red) not only contained the feature zones for PAAm hydrogel (blue) at 2750 cm^{-1} – 3500 cm^{-1} and 1300 cm^{-1} - 1500 cm^{-1} (as indicated by green rectangular frames), but also presented the

characteristic peak for GO sheets (black) at 1760 cm^{-1}. It demonstrates that GO sheets have been successfully covered the surface of PAAm hydrogels, forming a GO/PAAm composite structure.

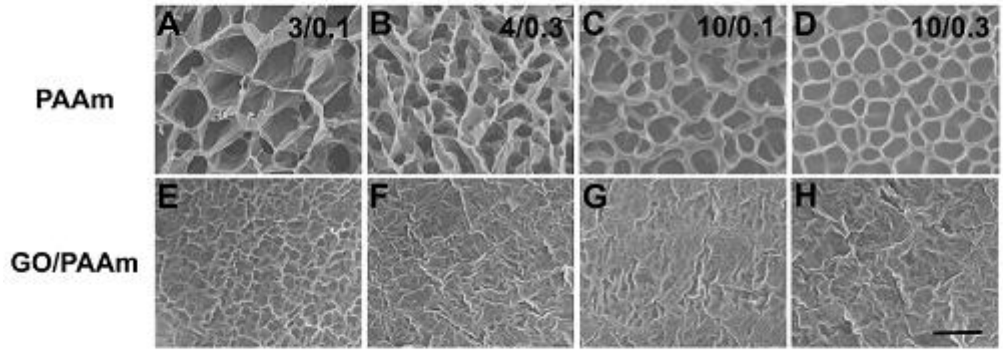

Figure S4. Fabrication of stiffness-tunable GO/PAAm composite scaffolds. (A-D) SEM images of PAAm hydrogels made with varying monomer/cross linker ratios. (E-H) SEM images of GO/PAAm composite scaffolds, corresponded to each PAAm hydrogels (scale bars, 10 μm).

As shown in Figure S4, the different hydrogels revealed significant differences in surface texture (or porosity). As the ratio of monomer-to-crosslinker from 3/0.1 to 10/0.3, the porous network structure of the PAAm hydrogel (freeze-drying) gradually changed, from a flexible, thin wall of hole to a tough, thick well. It was widely accepted that the variance of surface of different PAAm hydrogels resulted from the secondary effects. To address this issue, GO sheets were used to fabricate the GO/PAAm composite scaffolds to avoid the secondary effects. When coated with an optimum concentration of GO sheets, the composite scaffolds showed a typical wrinkled topography similar to GO thin films. Therefore, a stable GO/PAAm composite scaffold has been fabricated by PAAm hydrogel and GO sheets via a spin-coating method.

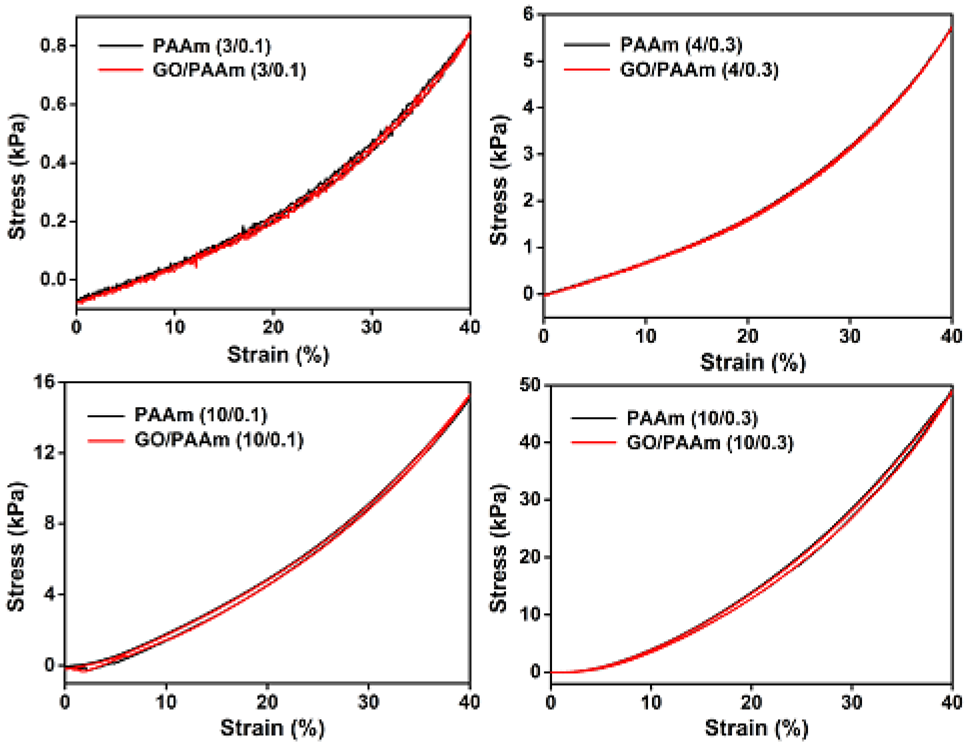

Figure S5. Stress-strain curves of PAAm hydrogels and GO/PAAm composite scaffolds with different formulations of acrylamide/bis-acrylamide: 3/0.1, 4/0.3, 10/0.1 and 10/0.3.

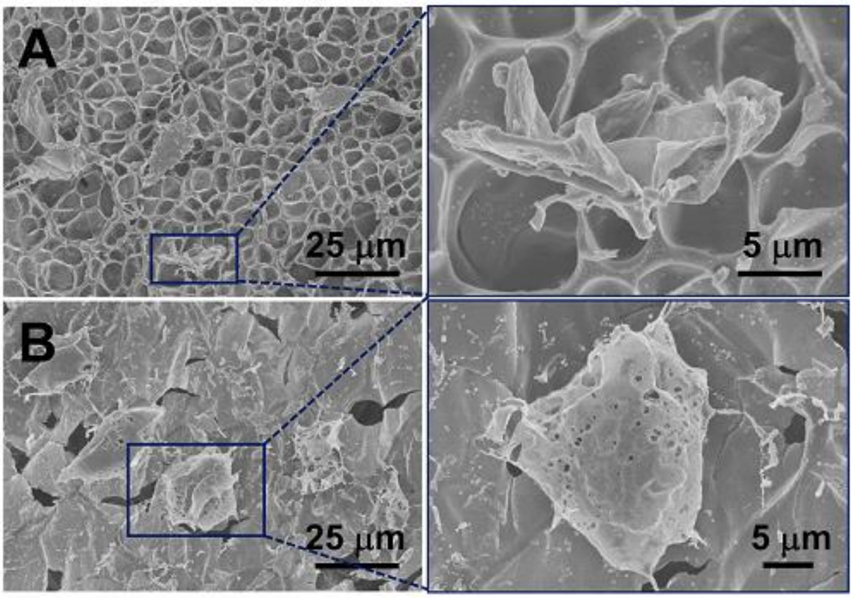

Figure S6. SEM images of HeLa cells on different substrates for 24h: (A) bare PAAm hydrogels and (B) GO/PAAm composite scaffold.

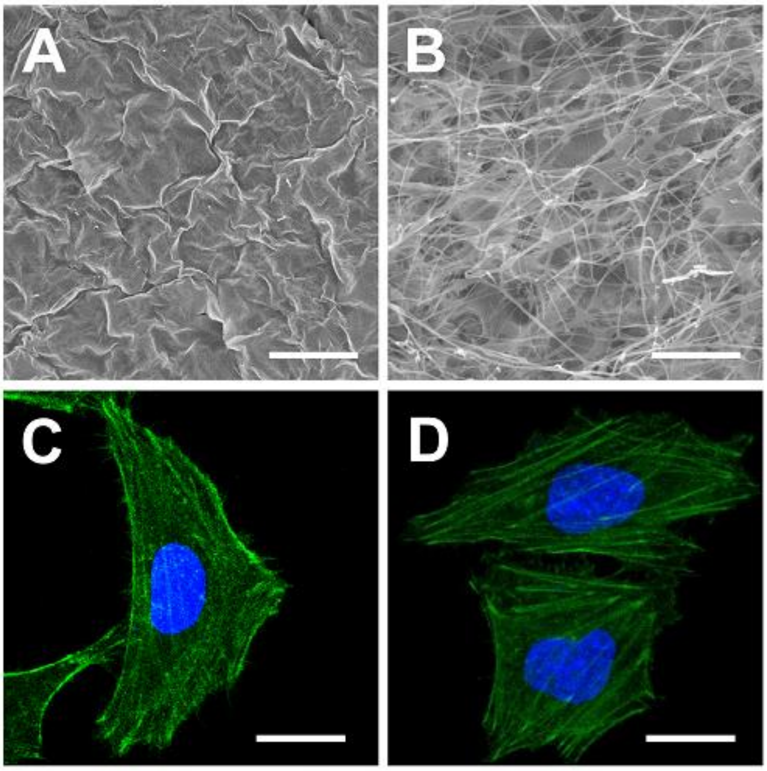

Figure S7. The surface morphology and cell behaviors for traditional collagen coated PAAm hydrogel and GO/PAAm composite scaffold. (A) SEM image of the morphology features of GO/PAAm composite scaffold (GO coated PAAm hydrogel, GO: 0.5mg/ml; PAAm: 32 kPa). (B) SEM image of the morphology features of collagen coated PAAm hydrogel (collagen: 0.1 mg/ml; sulfo-SANPAH: 0.2 mg/ml; PAAm: 32 kPa). Fluorescence image of HeLa cell on GO/PAAm composite scaffold (C) and collagen coated PAAm hydrogel (D).

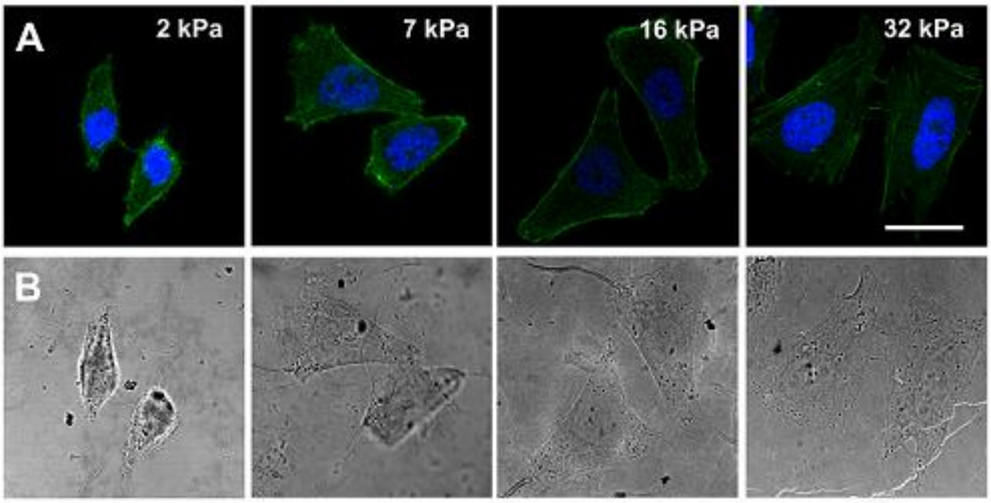

Figure S8. (A) Fluorescence images and (B) optical images of HeLa cells on the substrates with varied stiffness: 2 kPa, 7 kPa, 16 kPa, 32 kPa. Scale bar, 20 μm. Cells are stained with FITC-phalloidin and DAPI to visualize F-actins (green) and nuclei (blue) after culturing on the substrates for 24 h.

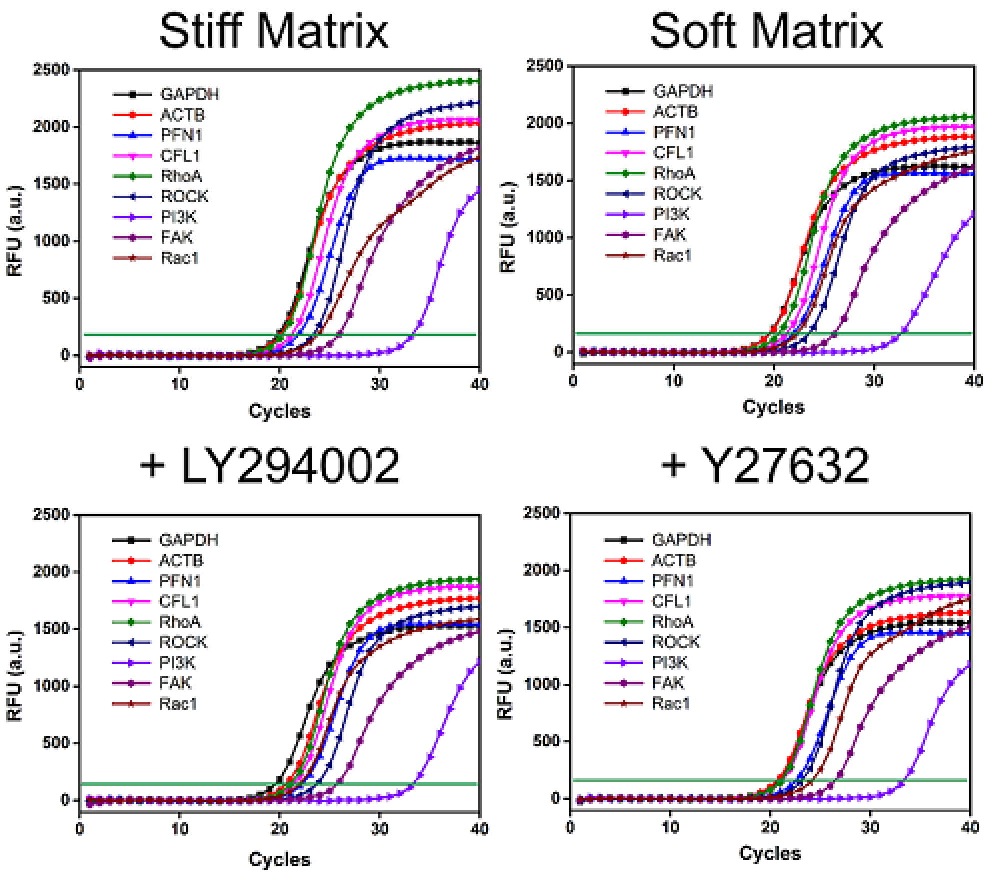

Figure S9. Expression analysis of target mRNAs in the HeLa cells under different stimulations by RT-qPCR: stiff matrix; soft matrix; LY294002 (inhibiting PI3K); Y27632 (inhibiting ROCK). The green horizontal line represents the threshold line.
